# EXAMINING THE PREVALENCE OF UNDIAGNOSED SLEEP APNEA AND ITS ASSOCIATION WITH BLOOD BIOMARKERS FOR ALZHEIMER’S

**DOI:** 10.1093/geroni/igad104.3321

**Published:** 2023-12-21

**Authors:** Mitchell Roberts, Erica Sappington, Michael Jaffee, Jeff Lowenkron, Paul Dagum, Jeffrey Iliff, Swati Rane Levendovszky, Carla VandeWeerd

**Affiliations:** UF Health Precision Health Research Center-The Villages, The Villages, Florida, United States; UF Health Precision Health Research Center-The Villages, The Villages, Florida, United States; University of Florida College of Medicine, Gainesville, Florida, United States; The Villages Health, The Villages, Florida, United States; Applied Cognition Inc, Redwood City, California, United States; University of Washington, Seattle, Washington, United States; University of Washington Medical Center, Seattle, Washington, United States; UF Health Precision Health Research Center-The Villages, The Villages, Florida, United States

## Abstract

Obstructive sleep apnea (OSA) prevalence increases with age, but is often undiagnosed. Recent findings suggest association between OSA and Alzheimer’s Disease (AD). OSA may impact alterations in amyloid-beta (Aß) protein possibly through interactions with the glymphatic system, thereby unraveling underlying AD mechanisms. In a crossover study conducted 06/22-04/23, older adults (55-65, mean 61.9) without diagnosis of OSA or cognitive impairment(N=33) completed MRIs and blood draws for AD-biomarkers across evening, morning, and midday time points. Polysomnography (PSG) using Philips Sleepware measured Apnea-hypopnea-index (AHI). Morning Trails A&B assessed cognitive function, and abeta40-42-42/40 levels were calculated using Immunoprecipitation-Mass-Spectrometry (IP-MS). Descriptive statistics, ANOVA, and linear regression were utilized. AHI scores averaged 10.5(SD:10.09), with 30% meeting ‘normal’ (0-5) and 52% mild (5-15) criteria. While not significant(p=.113), males(M:12.5,SD:9.7) averaged higher AHI scores than females (M:8.2 SD:10.3). AHI had a linear relationship with Abeta40 values(R2=0.18,F(2,33)=6.71,p=0.012). However, no significant association between AHI, Abeta42(p=0.09), and Abeta42.40(p=0.54) was found. Neither AHI sum scores [Trails A(p=0.09), Trails B(p=0.67)], nor severity rankings [TrailsA(p=0.74),TrailsB(p=0.14)] were significantly associated with Trails A&B performance. Elevated AHI was associated with higher levels of Aß40 but not cognitive test performance. Though implications are limited by sample size, findings suggest connection between OSA and initiation of AD processes, necessitating additional research.

